# Magnetic Resonance Imaging as a Tool for Monitoring Intratibial Growth of Experimental Prostate Cancer Metastases in Mice

**DOI:** 10.3390/mps6060118

**Published:** 2023-12-05

**Authors:** Junchi Huang, Mikael Montelius, Jan-Erik Damber, Karin Welén

**Affiliations:** 1 Sahlgrenska Center for Cancer Research, Department of Urology, Institute of Clinical Sciences, Sahlgrenska Academy, University of Gothenburg, 405 30 Gothenburg, Sweden; junchi.huang@gu.se (J.H.); jan-erik.damber@gu.se (J.-E.D.); 2 Department of Medical Radiation Sciences, Institute of Clinical Sciences, Sahlgrenska Academy, University of Gothenburg, 405 30 Gothenburg, Sweden; mikael.montelius@radfys.gu.se

**Keywords:** magnetic resonance imaging, prostate cancer, intratibial, xenograft, osteoblastic, bone lesion

## Abstract

Bone metastases cause morbidity and mortality in several human cancer forms. Experimental models are used to unravel the mechanisms and identify possible treatment targets. The location inside the skeleton complicates accurate assessment. This study evaluates the performance of magnetic resonance imaging (MRI) of prostate cancer tumors growing intratibially in mice. MRI detected intratibial tumor lesions with a sensitivity and specificity of 100% and 89%, respectively, compared to histological evaluation. Location and some phenotypical features could also be readily detected with MRI. Regarding volume estimation, the correlation between MRI and histological assessment was high (*p* < 0.001, r = 0.936). In conclusion, this study finds MRI to be a reliable tool for in vivo, non-invasive, non-ionizing, real-time monitoring of intratibial tumor growth.

## 1. Introduction

Bone metastasis is a major problem in several common human cancer forms, such as breast, prostate, and lung. Understanding the metastatic process and the growth in bone is the key to the inhibition of local, and often treatable, cancers from developing into lethal metastatic diseases. Magnetic resonance imaging (MRI) is routinely used for imaging in oncology. In prostate cancer, it is primarily used as a method for more precise diagnosis in combination with analysis of a prostate-specific antigen (PSA) in blood [1]. For bone metastatic disease, whole-body MRI is a tolerable and sensitive alternative to a prostate-specific membrane antigen (PSMA)-PET, especially in PSMA-negative tumors, and is useful when the detection of new lesions is important for treatment decisions [2].

Animal models of prostate cancer that metastasize to the skeleton are rare [3], and injecting cancer cells directly into the bone marrow cavity of the tibia provides a means to study the growth of cells with different or modified properties or effects of treatments on intratibial growth. However, the location and small size prevent manual measurements with calipers as is common for subcutaneous tumors, and proper characterization and volume assessments are limited, leading to considerable risks of both poor documentation of the tumor growth phase and the unnecessary suffering of mice due to extensive but undetected tumors in the bone marrow.

Fluorescence and luminescence technologies are frequently used to study tumors in animal studies, but the location inside the bone modifies and weakens the signals and may lead to incorrect comparative observations, especially during the monitoring of intratibial tumors prone to weakening or breaking the bone during progression. Computed tomography (CT) is often used in in vivo studies to monitor tumor growth. However, CT often requires exogenous contrast agents for adequate image contrast, and since CT relies on ionizing radiation, it might affect tumor growth and thus confound the interpretation of the treatment results. Using MRI, it is possible to acquire images of solid tumors with excellent contrast and resolution without the confounding effects of ionizing radiation [4].

In mice, where the tibial bone marrow cavity is approximately one millimeter wide, imaging is challenging. MRI has previously been demonstrated to accurately measure tumor sizes in soft tissues down to 0.02 g [5]. In the tibia of mice, however, tumors are expected to be even smaller when they are confined to the bone marrow cavity. Intratibially transplanted xenografts of osteosarcomas growing outside of the bone were readily imaged with MRI [6], as were the metastases of intracardially injected breast cancer cells [7]. Experimental intratibial prostate cancer tumors have indeed been successfully monitored using MRI, but these studies based their tumor volume estimates mostly on the extratibial part of the tumor in the surrounding bone muscle tissue [8,9].

**Figure 1:**
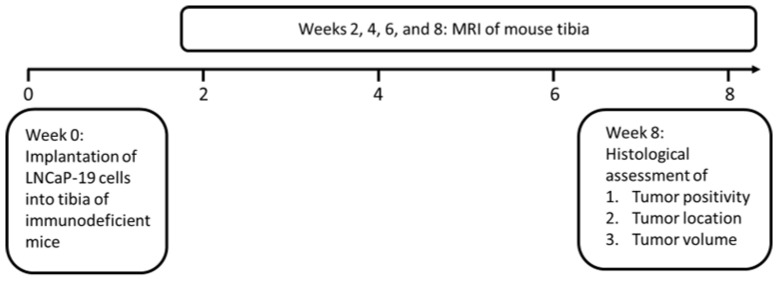
Schematic illustration of the experimental setup.

The aim of the present study was to evaluate the performance of MRI as a non-invasive technique to localize and estimate the sizes of intratibial prostate cancer tumors, monitor tumor growth longitudinally, and characterize their metastatic phenotype (Figure 1).

## 2. Material and Methods

### 2.1. Cell Culture

LNCaP-19 is an in-house osteogenic castration-resistant prostate cancer (CRPC) cell line developed from LNCaP that has been described previously [10,11]. LNCaP-19 cells were cultured in an RPMI 1640 medium (12-702F; Lonza, Switzerland) supplemented with glucose, a sodium pyruvate medium, and 10% charcoal–dextran-stripped serum ((CSS), Invitrogen). All cultures were complemented with 1% penicillin/streptomycin and confirmed to be mycoplasma free.

### 2.2. In Vivo Intratibial Tumors

This study was approved by the Gothenburg Ethical Committee on Animal Research (Permit Number 11672/2019). Intratibial injections were performed as previously described [11]. Briefly, Balb/c nude male mice (6–8 weeks old; Charles River Laboratories International, Inc., Wilmington, MA, USA) were anesthetized with isoflurane. The right leg was flexed, and a 29-gauge needle was inserted with a drilling motion via the knee joint into the proximal end of the tibia. 1 × 10^6^ LNCaP-19 cells in 7 µL Matrigel (BD Bioscience, Bedford, MA, USA) were injected into the bone marrow cavity. The mice were castrated directly before cell implantation to mimic the situation of androgen deprivation in humans. The experiment ended after 8 weeks. For histological evaluation, the tibias were dissected and fixed in formalin, decalcified in EDTA for three weeks, and embedded in paraffin.

### 2.3. Magnetic Resonance Imaging

At weeks 2, 4, 6, and 8 after the implantation, the tumors were imaged using a small-animal 7T MRI system (BioSpec 70/20AS AVANCE 1; ParaVision software version 5.1; Bruker BioSpin MRI GmbH, Ettlingen, Germany) equipped with a 72 mm volume transmit coil and a 4-channel array receiver coil (RAPID Biomedical GmbH, Rimpar, Germany).

Animals were anesthetized using 4–5% isoflurane (Isoba vet., Schering-Plough Animal-health, Denmark) in air before they were positioned headfirst on their right on the animal holder, where anesthesia was maintained during the imaging experiment using ~2–3% isoflurane in oxygen-enriched air. A circulating warm water system was used to maintain body temperature, and respiration was monitored using a pressure-sensitive pad (SA Instruments, Inc., Stony Brook, NY, USA).

The Bruker tripilot localizer scan was run to verify the position of the animal and plan the position of a transversal and a sagittal image volume of the mouse tibia, thus producing cross-sectional images of the tibia and images parallel to its long axis, respectively. T_2_-weighed, fat-suppressed, 2-dimensional rapid acquisition with relaxation enhancement (RARE) sequences were used for imaging, with saturation slices to suppress unwanted signal, e.g., from subcutaneous fat close to the coil elements. Imaging parameters are listed in Table 1.

Tumor volumes were calculated using an in-house developed graphical user interface in Matlab (R2019b, The MathWorks Inc., Natick, MA, USA). In brief, the MR images were presented to the operator, with the possibility to manually adjust display settings for optimal tumor visualization. A region of interest (ROI) was then created on each section of the MRI volume by manually tracing out the tumor border. Pixels intersecting the tumor border were excluded to avoid partial volume effects. The tumor volume was then calculated by summing up the number of pixels within all ROIs and multiplying it by the voxel dimensions, including the slice gap.

### 2.4. Histological Evaluation

Serial, 4 µm thick sections of the tibia, covering the entire extent of the tumor, were acquired using a microtome. Sections were pre-heated at 60 °C for 10 min, deparaffinized and rehydrated in ethanol, counterstained with Mayer’s hematoxylin, and dehydrated and mounted in Pertex® (Histolab, Gothenburg, Sweden).

Tumor volumes were estimated based on serial sections covering the entire tumor, as previously described [12]. Briefly, by assuming an ellipsoidal tumor shape and measuring the major (a) and minor (perpendicular, b) semi-axes, section areas (A) were calculated as A = a × b × π. Section volumes were then calculated by multiplying the area by the section thickness, and the final tumor volume was calculated by summing all section volumes. 

### 2.5. Statistics

Statistical calculations were performed using the SPSSv20 software package (SPSS, Chicago, IL, USA). The method authentications were using Pearson correlation and Bland-Altman analysis. Results with *p*-value < 0.05 were considered statistically significant.

## 3. Results

### 3.1. MRI Performance for Tumor Location and Phenotype Definition

**Figure 2:**
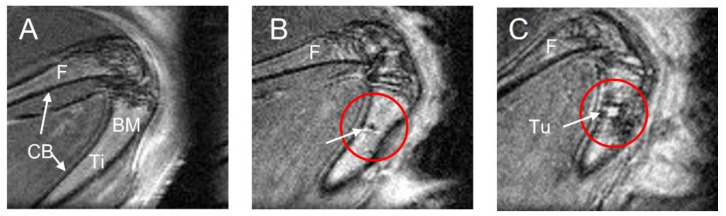
Tibia and tumor phenotype in MRI. (**A**) Femur (F) and tibia (Ti) with tumor-free bone marrow cavity (BM) and cortical bone (CB); (**B**) tibia with a possible sign of the injection as scar tissue or blood marked with an arrow in a red circle; (**C**) intratibial tumor (Tu) with high signal intensity, possibly necrosis/inflammation, marked with an arrow in a red circle.

Compared to a healthy tibia where bone marrow is visible as a homogenous MR signal intensity (bright, Figure 2A) enclosed by the cortical bone (dark, Figure 2A), an abnormal hypointense (darker) area in the tibia can be seen at week 4, a possible sign of scar tissue on the injection site or accumulation of blood (red circle, Figure 2B). In these T_2_-weighted images, fluid accumulation (high signal intensity) possibly caused by inflammation or necrosis can be detected in tumors (Figure 2C). It should be noted that the image acquisitions were optimized to visualize the tibia, which required tradeoffs in image quality for areas outside these regions of interest. This is why, e.g., artifactually high signal from subcutaneous fat or signal voids from saturation bands (e.g., regions on the right in Figure 2) were considered acceptable.

**Figure 3:**
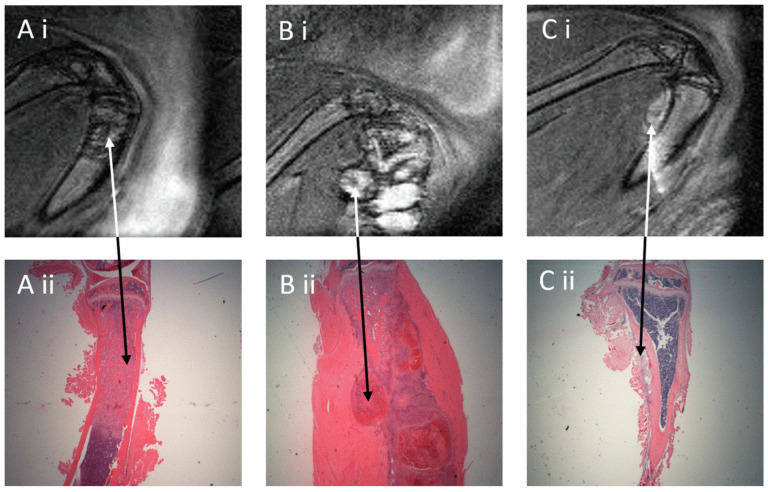
Comparison of MRI and histological sections of the same tibia sample. Panels show representative pictures of three tibia samples in MRI (**i**) and histological sections (**ii**); original magnification 4×, and arrows in paired pictures point corresponding area of the same tibia. (**A**) Osteoblastic structure represented by a speckled appearance of a newly formed trabecular bone; (**B**) tumor with osteolytic property causing degradation of the cortical bone; (**C**) tumor growth outside the tibia. Note: the histological section of the tibia in C is performed at a different angle, unfortunately missing a large part of the bone area, making the position of the tumor appear different.

In large tumors, the phenotype could be distinguished as osteoblastic represented by a speckled appearance of newly formed trabecular bone (dark) within the tumor tissue (white) (Figure 3A(i)). Osteolytic activity or tumor growth may cause breakage of the bone and allow tumor growth outside the tibia (Figure 3B(i)). Occasionally, no tumor was observed within the tibia but could be localized outside of the tibia, probably due to erroneous injection (Figure 3C(i), unfortunately, there is an artifact in this image, adding uncertainty to the tumor definition). MRI displayed a good agreement with histological images in the same areas (Figure 3A(ii), B(ii), C(ii)), and showed the location and structure of the tumors accurately.

### 3.2. MRI Performance in Evaluating Tumor Positivity

To validate the accuracy of MRI to define tumor positivity, the outcomes in 41 tibia samples at the end stage (week 8) were analyzed in both MR images and histologic sections. In the histological sections, 13 were defined as tumor positive and 28 were tumor negative. With MRI, all 13 positive cases were detected, giving a sensitivity of 100%. Of the twenty-eight tumor-negative cases defined by histology, MRI correctly classified twenty-five as negative, but three contained structures that were falsely defined as tumors in the MR images, giving a specificity of 89%. In the earlier stage (week 4 or 6), three mice showed possible tumors, while no sign of tumors was visible at week 8 in the same animals, in accordance with histological sections at week 8. 

### 3.3. MRI Performance in Assessing Tumor Growth

**Figure 4:**
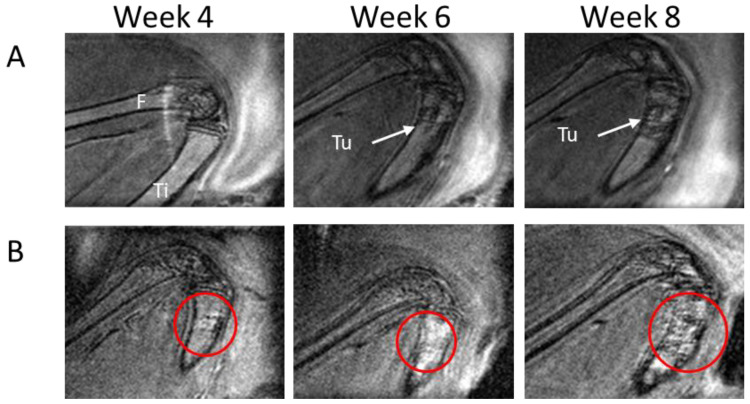
Tumor growth monitored with MRI. Representative images of tumor growth at the 4-, 6-, and 8-week MRI time points of (**A**) small tumors; (**B**) large tumors. F = femur, Ti = tibia, Tu = tumor. In B, red circles indicate tumor location.

**Figure 5:**
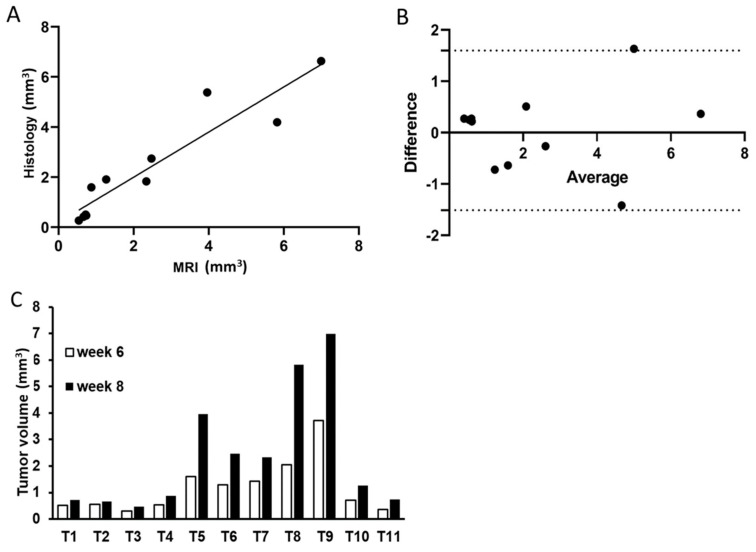
Evaluation of tumor volume estimation by MRI. (**A**) Correlation of tumor volume estimation by MRI and histological sections (Pearson correlation *p* < 0.001). (**B**) Bland–Altman analysis of MRI and histology volume estimates with a 95% CI (dashed lines). (**C**) Illustration of the MRI determined volumes of individual tumors at week 6 and week 8.

Tumors were monitored with MRI in weeks 2, 4, 6, and 8 to assess intratibial tumor growth. We identified progressive growth in the tibia both for smaller (Figure 4A) and larger (Figure 4B) tumors. To further investigate the reliability of using MRI for tumor volume assessment, the estimated tumor volumes based on MRI and histology at week 8 were compared. The methods correlated well (correlation coefficient (r) = 0.936, *p* < 0.001, Figure 5A), with the biggest deviation observed for the largest tumors (Figure 5B). In line with this, the MRI data at week 6 and week 8 were compared and showed, as expected, tumor growth in all tumors (Figure 5C), with an average increase in volume of 84% (median: 77.8%, min: 21.0%, max: 184%).

## 4. Discussion

Intratibial xenografts are a good model for studying prostate cancer growth in bone. However, one limitation is the difficulty of monitoring tumor progression in vivo since the location is not readily accessible for volume assessment. In the present study, we evaluated the performance of MRI as a method to monitor tumor growth in mouse tibia.

We have previously used MRI to successfully follow orthotopic prostate cancer xenografts [13], and the present study demonstrates its usability for the intratibial setting. The MR images at the last time point were compared with corresponding histological images, and the concordance was good. All tumors detected in the sections by histology were also identified with MRI. In addition, in some cases, MRI analysis detected suspected undefined tumors at earlier time points, which were not confirmed as tumors using histology at the last time point. This uncertainty makes the ratio of false positive MRI-detected tumors difficult to interpret since it is possible that MRI in these cases detected true tumors that later regressed. 

We could accurately localize intratibial tumors on MRI, and volume estimates correlated well with histological volume estimates. The largest deviations were found for the largest MRI-based tumor volumes, where histological evaluation tended to give a smaller volume estimate. This bias may be due to an underestimation of volumes based on histology since accurate sectioning of bone largely disrupted by the extensive tumor growth is difficult, and some areas may be lost.

The volume comparisons were performed during the experiment when histological samples could be attained, and, therefore, no conclusions can be made regarding the accuracy of volume estimates at the early establishment phase when other biological processes might be ongoing in the bone microenvironment, possibly interfering with tumor border definition. 

We were able to distinguish phenotypic traits, such as a speckled appearance reflecting the highly branched growth of trabecular bone, seen as “islands” on histological sections, characteristic for the osteoblastic growth of LNCaP-19 [11], as well as other osteoblastic tumor models [14,15]. This is of interest when investigating different environmental or pharmaceutical interventions for their effect on tumor phenotypic development.

The main limitation of the methodology used is the difficulty to correctly evaluate very small lesions. We observed some suspected small tumors at an early time point, which were, however, not confirmed as tumors at a later stage. We cannot conclude whether these observations were in fact tumors that failed to establish or regress or some other non-tumor related reaction, such as temporary inflammation after the injection.

CT is commonly used in in vivo studies of tumors, but the technique requires exogenous contrast agents for better visualization [16]. The relatively high radiation dose required for high-resolution images could affect the tumor activities, which could confound the interpretation of the results. Using MRI, with its superior soft tissue contrast (as required for the tumors in our study), we could acquire images without the use of radiation or contrast agents. 

One limitation of this study is its descriptive nature, making it impossible to determine whether MRI gives more accurate observations than other imaging methods. The present study describes the performance of MRI regarding some phenotypical characteristics, location, and monitoring of growth, but it is inconclusive with regards to whether it should be the method of choice for non-invasive, longitudinal assessments of intratibial prostate cancer tumor models. Future studies that compare MRI and histology at earlier time points should be conducted to describe the performance of MRI regarding the very early, small tumor lesions. Other interesting MRI techniques should also be investigated to enhance the specificity of the method to characterize tumor biology and physiology. For example, diffusion-based MRI techniques could be used to study tumor vascularity and cell density based on models, such as the intravoxel incoherent motion (IVIM) model, where the MRI signal is made sensitive to the molecular motion of vascular (blood) and tissue water. Since such techniques can be added to the MRI examination with only an extended imaging time, they are very suitable for preclinical studies and may help characterize tumor tissue both spatially and longitudinally. However, more optimization is required since advanced MRI techniques tend to become more sensitive to, e.g., susceptibility differences between air and soft and hard tissue borders, which may result in problematic image artifacts. For translation to humans, the techniques also need optimization regarding scan time since MRI examinations are already relatively long and expensive in human applications. 

MRI provides several advantages for its use in studies of intratibial tumors in mice. First, it allows longitudinal assessment during tumor progression. Second, the excellent soft tissue contrast and resolution allow visualization and volume quantification within the not easily accessible intratibial location, which is of utmost importance for understanding tumor progression. Third, it confers minimal interference with the tumor progression and the health of mice, which are important aspects since obtruding factors could impair the conclusion of any observed effects due to ethical aspects, respectively. 

In conclusion, the present study demonstrated the potential of MRI for analysis of bone metastases in mice, making MRI a valuable tool for in vivo tumor research, especially at anatomically inaccessible locations.

## Figures and Tables

**Table 1:** MRI sequence parameters.

Imaging Parameters	Transversal	Sagittal
Repetition time (ms)	2500	2700
Effective echo time (ms)	28	18.5
Turbo factor	6	4
Number of averages	4	20
FOV (read × phase) (mm)	19 × 13	16.2 × 13.5
Matrix size (read × phase)	120 × 80	134 × 160
Pixel size (read × phase) (mm)	0.16 × 0.16	0.12 × 0.09
Number of slices	16	9
Slice thickness (mm)	0.5	0.23
Slice gap	0.5	0.1
Fat suppression	yes	yes
Respiratory triggering	no	no
Scan time	2 min 10 s	36 min

## Data Availability

Data are contained within the article.
